# Editorial: Identification of neurological biomarkers for neurodegenerative disorders

**DOI:** 10.3389/fneur.2026.1973360

**Published:** 2026-09-11

**Authors:** K. Jayasankara Reddy, Sruthy Nair

**Affiliations:** Department of Psychology, Christ University, Bangalore, India

**Keywords:** cognitive impairment, cognitive screening, multimodal biomarkers, neurodegenerative disease, neuroimaging, neurological biomarkers

Neurodegenerative diseases are a heterogeneous group of disorders characterized by the progressive loss of neurons in the nervous system. This progressive disruption in the structure and function of neural networks, combined with their inability to renew themselves due to their terminally differentiated nature, results in the breakdown of core sensory, cognitive, behavioral, and motor functioning (1).

Neurodegenerative diseases are characterized by distinct biological, cognitive, and behavioral changes. While traditional diagnostic frameworks, often acting as late-stage detection systems, rely on the exploration of behavioral symptoms and cognitive profiling, biological markers matter because they bridge the gap between the initiation of irreversible structural damage and the onset of observable symptoms, a gap that behavioral and cognitive assessments alone may not adequately address.

The effectiveness of disease-modifying therapies may depend partly on interventions at earlier stages of the disease, highlighting the need for biomarkers capable of identifying pathological changes before substantial clinical symptoms arise. Against this backdrop, this Research Topic brings together studies that advance biomarker discovery and clinical translation across neurodegenerative disorders.

This Research Topic comprises 13 articles, including six original research articles, two systematic reviews, two reviews, two mini reviews, and one brief research report. The articles focus on current approaches to the biomarker discovery and its implications in neurodegenerative disorders.

Isik et al. compared retinal and optic nerve head microvasculature using OCT-A in asymptomatic HTLV-1 carriers and HAM/TSP patients. Although no significant differences emerged, reduced peripapillary flux index in HAM/TSP suggested its potential as a biomarker of disease progression warranting further investigation. The study by Renga et al. used multimodal MRI to compare ALS patients with healthy controls, revealing widespread network disruption and altered structural-functional coupling. These findings support ALS as a network-level neurodegenerative disorder and highlight multimodal connectomic measures as potential biomarkers. Xu et al., investigated if the combined triglyceride glucose (TyG) and non-high density lipoprotein cholesterol (non-HDL-C) could predict early cognitive impairment following acute ischemic stroke. It was also found that higher TyG and non-HDL-C levels were independently associated with an increased post-stroke cognitive impairment, with their combined use associated with improving predictive accuracy.

Santacesaria et al. found that patients with Parkinson's disorder (PD) without mild cognitive impairment (MCI) showed normal prospective memory performance despite distinct oscillatory patterns, suggesting compensatory neural mechanisms. Time-based prospective memory involved increased alpha, theta, and beta power, whereas event-based memory showed reduced power, indicating differing attention-control strategies despite preserved performance.

The study by Chen et al., found that a higher MRI derived global cerebral small vessel disease burden is associated with hippocampal sclerosis, higher is the cognitive impairment, and more advanced stages along the Alzheimer's disease (AD) continuum, suggesting it may serve as a useful imaging biomarker for AD. The study on multiple system atrophy (MSA) by Huang et al. found that low baseline serum total cholesterol independently predicted higher mortality, indicating serum cholesterol may serve as a prognostic biomarker for survival in MSA.

The meta analysis by You et al., found that a higher neutrophil to lymphocyte ratio (NLR) is linked to the presence and higher burden on cerebral small vessel disease, especially the ischemic markers. This suggests that NLR may serve as inflammatory biomarkers for ischemic cerebral small vessel disease, requiring further investigation through longitudinal studies. The review article by Altuna examines the shift in early cognitive screening following the introduction of disease-modifying therapies for Alzheimer's disease. While traditional paper tools remain practical, they lack sensitivity and are prone to biases. Digital cognitive phenotyping offers an accessible approach to stratifying early risk guiding further testing and diagnosis.

The systematic review by Yang et al. evaluates the use and predictive value of Galectin-3 as a blood biomarker for early cognitive development. Elevated peripheral Gal-3 was associated with cognitive impairment. The authors concluded that Gal-3 may be an accessible and non-invasive biomarker for the early detection of vascular-related cognitive decline and MCl, however, its further role in AD is yet to be clarified. Guo et al. focuses on the shift toward multimodal biomarkers; neuroimaging, blood/CSF proteins, genetics, digital tracking and clinical tests; for early detection of MCI in PD. AI assisted approaches may help overcome the low sensitivity of traditional paper tests to enable precise risk understanding and timely interventions to delay progression to dementia. Li and Thirupathi highlights the dual nature of Interleukin-6, a cytokine that acts as a pro-inflammatory agent in the brain and a beneficial myokine that promotes neuroplasticity. Chronic inflammation elevates harmful IL-6-trans-signaling, whereas exercise typically triggers the classical IL-6 signaling pathways that support neuroprotective factors.

Getahun et al. found that declining retinal total macular volume (TMV) in 11 RVCL-S patients over 2 years was associated with worsening brain pathology. Using OCT and MRI, the study suggests retinal TMV may serve as an accessible biomarker for monitoring cerebral disease progression. Within the evaluated biomarkers, Deng et al. found that CSF p-tau217 had the highest standalone diagnostic accuracy for Alzheimer's disease (95% sensitivity, 94% specificity). The Aβ42/p-tau181 ratio showed the best combined performance, supporting a panel of p-tau217 and Aβ42/p-tau181 for early AD diagnosis.

Taken together, the Research Topic point toward an important shift in identification and monitoring of neurodegenerative disorders where biomarkers are moving toward more integrated and multimodal approaches. Across the included studies, retinal measures, neuroimaging markers, blood and cerebrospinal fluid markers, electrophysiological measures, digital cognitive phenotyping, and clinical characteristics provide complementary information about disease related changes. These approaches have the potential to extend clinical assessment by identifying biological changes that may precede or accompany clinically observed impairments. A common message emerging from this Research Topic is that a multimodal approach provides a comprehensive characterization of disease risk, progression and prognosis.

Despite these advances, important gaps remain before many emerging biomarkers can be translated into routine clinical practice. Several studies rely on relatively small samples, and differences in study populations, acquisition protocols, analytical methods, and outcome definitions can limit comparability and generalizability. The high cost and limited availability of advanced neuroimaging and other specialized technologies may further restrict their implementation, particularly in resource-limited settings. Standardized protocols, larger and more diverse longitudinal cohorts, independent validation, and clinically meaningful outcome measures will therefore be essential for establishing the reliability and utility of candidate biomarkers.

An important priority for the field is consequently to move beyond biomarker discovery toward rigorous validation and implementation. Future studies should determine whether biomarkers provide incremental clinical value over established assessments and whether their use improves diagnostic accuracy, prognostic stratification, treatment selection, or monitoring of therapeutic response. Multimodal biomarker panels, combined with appropriately validated artificial intelligence approaches, may offer an important avenue for achieving this goal. However, such approaches should complement rather than replace clinical expertise, and their implementation will require attention to interpretability, reproducibility, accessibility, and ethical considerations.

Overall, this Research Topic highlights substantial progress in the identification of biomarkers for neurodegenerative disorders, while also demonstrating specific biomarkers at different stages of clinical development. Some approaches remain exploratory and require replication and longitudinal validation, while others show greater potential for clinical implementation. Future research needs to prioritize standardization, accessibility and clinical utilization of biomarkers.
